# The Relationship Between Tranquility Level and Professional Satisfaction in Nurses

**DOI:** 10.1155/jonm/5053232

**Published:** 2025-04-09

**Authors:** Burcu Genç Köse, Ayşe Gümüşler Başaran, Bahar Kefeli Çol

**Affiliations:** ^1^Faculty of Health Sciences, Recep Tayyip Erdogan University, Rize, Türkiye; ^2^Güneysu School of Physical Therapy and Rehabilitation, Recep Tayyip Erdogan University, Rize, Türkiye

**Keywords:** hospital, nurse, professional, satisfaction, tranquility of mind

## Abstract

**Aim:** The study was conducted to determine the level of tranquility and professional satisfaction in nurses working in two different cities.

**Background:** Peace of mind, the expectation of all individuals throughout life, is essential in the nursing profession, which performs patient care and treatment. The satisfaction of the service and care provided to others is related to the mental peace and satisfaction of the nurse.

**Methods:** The study is a descriptive type. The study was conducted in three hospitals in two provinces. No sample selection was made, and data were collected from 546 nurses who agreed to participate in the study. The nurse recognition form, tranquility scale, and professional satisfaction scale were used to collect the data.

**Results:** Nurses' tranquility and professional satisfaction levels were moderate. The level of tranquility of postgraduate and single nurses was significantly higher. The level of tranquility and professional satisfaction was significantly higher in nurses with adequate income, professional experience, years of working in an organization of 11 years or more, and a managerial position. The professional satisfaction score was significantly higher in nurses working at “*X*” training and research hospital. A positive correlation was found between the tranquility and professional satisfaction scales.

**Conclusion:** Sufficiency of income level, professional experience, continuity in the institution, and working in a managerial position positively affected the level of tranquility and professional satisfaction. In this context, institution managers should implement policies to ensure organizational continuity.

**Implications for Nursing Management:** The positive relationship between tranquility and professional satisfaction emphasizes the importance of increasing nurse tranquility in institutions. Regulation of working environments affects nurses' levels of tranquility, and regular implementation of practices such as recognition and promotion will increase professional satisfaction and tranquility and will positively reflect on the quality of care. In metropolitan cities where professional satisfaction is higher, conducting studies that include external and internal factors are recommended to reveal the reason for the difference.

## 1. Background

Nursing is an important professional group recognized within the health care system and around the world [1]. It is known that nurses with intense workloads have moderate stress levels [2]. Tranquility, which can be affected by stress and workload, refers to an individual's inner tranquility, balance, or discomfort [3]. It is also defined as mental well-being, tranquility, and emotional calmness [4]. The Turkish equivalent of tranquility, which is the English word “tranquility,” is defined as “calmness and tranquility” [5]. As a concept, it is rooted in the Greek Philosophy and was born from the idea of happiness [4]. Tranquility is a universal life experience for individuals. Feeling tranquility brings quality of life and is an essential concept for the nursing profession [6].

Professional satisfaction, which is affected by intrinsic factors such as success, recognition, and progress, is also affected by extrinsic factors such as working conditions, workload, interpersonal relationships, and stress [7]. Professional satisfaction is a complex phenomenon researched by many disciplines [8] and is a concept that improves the quality of care provided by nurses and reveals occupational well-being [8, 9]. Looking at working conditions, one of the external factors affecting professional satisfaction, it was found that there was a significant relationship between professional satisfaction and burnout in nurses working in specialized units (e.g., neonatal units) [10]. Similarly, in a systematic review, it was hypothesized that stress factors have a significant impact on professional satisfaction in oncology units [11].

The communication and behaviors of nurses with each other in the work environment determine the tranquility in the work environment [12], and a tranquility working environment affects the motivation of nurses [13]. Tranquility, which represents these feelings, is the spiritual expectation that individuals express the most in their daily life cycle [3, 14]. While it was determined that nursing students considered tranquility among the important values related to being healthy [15], it is stated that workplace tranquility positively affects the performance of the individual [16]. It is indicated in the literature that being tranquility is one of the characteristics of a resilient nurse [17]. Tranquility in the work environment is a factor that is the basis for working individuals to support each other and stand firm in the face of complex and tense environments [18]. A tranquil working environment for nurses will bring effective care and quality health services.

Another concept that affects quality and practical working life is professional satisfaction, which is the emotional result that emerges in the individual's mind after evaluating their job [19]. Although many studies on job satisfaction in nurses are in the literature [20–22], professional satisfaction data are limited. Although it is known that excessive workload leads to dissatisfaction in health professionals [23], it was found that nurses had low levels of professional's satisfaction in their first year [13]. In a study conducted in a university hospital during COVID-19, nurses' professional satisfaction was moderate [24]. The literature has reported that increased professional satisfaction positively affects the quality of care in nurses who adopt evidence-based practices and knowledge [8]. At the same time, high occupational satisfaction reveals the inner well-being of the individual and appears as a determinant of decisions such as the intention to leave the job [25].

While social relations within the organization positively affect professional satisfaction [7], it is stated in the literature that happiness and tranquility may increase in individuals who are satisfied with their profession and do the job they love [26]. The number of studies investigating the level of tranquility in nurses is insufficient, and no study has been found to examine its relationship with professional satisfaction.

## 2. Methods

### 2.1. Aims

This study, which will contribute to the literature, aimed to determine the levels of tranquility and professional satisfaction in nurses working in two different cities and the relationship between them.

- Do nurses working in different cities have the same tranquility?
- Do nurses working in different cities have the same level of professional satisfaction?
- Is there a relationship between nurses' tranquility level and professional satisfaction?

### 2.2. Study Setting and Sampling

This descriptive study was conducted in April–May 2023. The universe of the study consisted of a total of 1260 nurses working in three hospitals in two different cities in Turkey. Hospital “*X*,” located in a metropolitan area, is a 530-bed Training and Research Hospital serving approximately 45,000 inpatients annually. “*Y*” Hospital, located within the scope of a small city, is a 500-bed training and research hospital serving approximately 40,000 inpatients annually. Hospital “*Z*,” located in a small city, is a state hospital with 300 beds, serving approximately 14 thousand inpatients annually.

The number of nurses working in the institutions was 600 in “*X*” Training and Research Hospital, 310 in “*Y*” Training and Research Hospital, and 250 in “*Z*” State Hospital. No sample was selected, and the study was completed with 546 nurses who agreed to participate.

### 2.3. Data Collection

The purpose of the study was explained to the nurse managers, and their voluntary informed consent was obtained. The study data were collected using the nurse recognition form, tranquility scale, and professional satisfaction scale. The forms and scales were prepared on paper and distributed to the nurse managers. They were asked to complete those by themselves and were given 1 week to do so.

#### 2.3.1. Nurse Recognition Form

The researchers' questionnaire consisted of ten questions, including age, gender, educational status, marital status, income status, institution of employment, years of professional experience, years of employment in the organization, position, and working style.

#### 2.3.2. Tranquility Scale

Demirci and Ekşi developed the scale with one dimension and eight items. It is scored as (1) not at all suitable for me, (2) not suitable for me, (3) somewhat suitable for me, (4) quite suitable for me, and (5) entirely suitable for me, and Items 5 and 6 are reversed. The scale's items include statements to assess one's state of feeling tranquility, feeling secure, state of harmony in one's life, state of tension, state of balance, state of uncertainty or confusion, and state of being at peace with oneself. The lowest score on the scale is eight, and the highest is 40. Depending on the results of the exploratory factor analysis that aims to evaluate the validity of the Peace Scale, it was seen that the scale had a one-dimensional structure with a score of 3226 eigenvalues, explaining 40,328% of the total variance and consisting of 8 items. The factor loadings of the measured items range from 0.55 to 0.71. Correlations of adjusted item-total scores of the items vary between 0.42 and 0.56. The fit index values obtained as a result of the confirmatory factor analysis were found to support one-dimensional structure in the first sample (*χ*^2^ [20, *N* = 450] = 58.48, *x*^2^/sd = 2.93, *p* < 0.001, CFI = 0.97, NFI = 0.96, NNFI = 0.96, SRMR = 0.041, RMSEA = 0.065) and in the second sample (*χ*^2^ [20, *N* = 450] = 74,05, *x*^2^/sd = 3,67, *p* < 0.001, CFI = 0.96, NFI = 0.95, NNFI = 0.94, SRMR = 0.045, RMSEA = 0.078). The scale's Cronbach alpha internal consistency coefficient was calculated as 0.78. As a result of these values, it was stated that the scale can be used in studies conducted in Turkey [3]. In this study, Cronbach alpha value was 0.748.

#### 2.3.3. Professional Satisfaction Scale

Developed in 1999 by Kuzgun, Sevim, and Hamamci, the scale is a five-point Likert-type with 20 questions. It has two subdimensions: Compliance with Qualifications (1, 2, 3, 4, 6, 8, 9, 10, 14, 15, 17, 18, and 19) and Willingness to Evolve (5, 7, 11, 12, 13, 16, and 20). The questions are scored between “5 = *always*” and “1 = *never*,” while six questions (4, 9, 10, 11, 14, and 19) are reverse-scored. The items of the scale include statements to evaluate the person's willingness to choose their profession again, to recommend their profession to others, to come to work enthusiastically, to retire early, to come to work eagerly, to increase their professional knowledge, to want to move to another job, the suitability of their job to their abilities, and to follow the publications related to their profession. The minimum score is 20, and the maximum score is 100. It is said that the higher the score, the higher the professional satisfaction. The validity of the scale was determined by factor analysis. As a result of the factor analysis, it was seen that the scale items were divided into two factors. The two factors of the scale determined by factor analysis were named Factor 1 as Compliance with Qualifications and Factor 2 as Willingness to Evolve, taking into account the characteristics of the items. The total variance explained by the two factors is 48.6%. Of this, 36.4% comes from the first and 12.2% from the second factors. Kuzgun et al. determined the Cronbach alpha value to be 0.90. As a result of these values, it is stated that the scale is intended to determine the occupational satisfaction of people working in any job [27]. In this study, the Cronbach alpha value was 0.855.

### 2.4. Statistical Analysis

The SPSS22 package program was used for the statistical analysis of the data. Descriptive data were expressed as percentage, mean, and standard deviation. Student's *t*-test, ANOVA, Pearson correlation analysis, and linear regression analysis were performed. In correlation analysis, 0–0.39 was considered a weak correlation, 0.40–0.69 a moderate correlation, 0.70–0.89 a strong correlation, and 0.90–1.00 a robust correlation. The significance value was accepted as *p* < 0.05.

### 2.5. Ethical Considerations

The research was carried out according to the guidelines in the Declaration of Helsinki and the ethical rules specified in the Ethics Committee of Recep Tayyip Erdogan University between April–May 2023. The study was conducted after obtaining institutional permissions from Recep Tayyip Erdogan University Social and Humanities Ethics Committee (2022\268) and the Health Directorates of both provinces (16.03.2023 and 02.01.2023). Nurses approved the informed consent form before responding.

## 3. Results

The mean age of the nurses participating in the study was 31.49 ± 7.54, and 83.2% were female. 90.8% had associate's/undergraduate degrees, 52.6% were single, and 53.1% had partially sufficient income. 37.5% worked at “*Y*” Training and Research Hospital, 30.2% at “*Z*” State Hospital, and 32.2% at “*X*” Training and Research Hospital. 68.7% have 0–10 years of professional experience, while 75.8% have 0–10 years working in their current institution. 78.8% of the nurses work day and night, and 86.4% work in the nurse position. Descriptive data are shown in Table 1.

The mean scores of the participants from the tranquility scale, professional satisfaction scale, and its subdimensions are shown in Table 2. The mean total score of the tranquility scale was 28.10 ± 5.20, while the mean score of the professional satisfaction scale was 59.71 ± 11.59.

The analysis of the participants' tranquility scale with independent variables is given in Table 3. There was a significant difference between the educational level of the nurses and their level of tranquility (*p* = 0.017). In the post hoc analysis, the level of tranquility of postgraduate nurses was significantly higher than that of high school graduates. When we looked at marital status, the level of tranquility was considerably higher in single nurses (*p* = 0.008). In addition, income status also created a significant difference (*p* = 0.000). As a result of post hoc analysis, all of them were significantly different. Those who found their income level as sufficient had a substantially higher level of tranquility than the other two groups, and those who found their income level as partially sufficient had a significantly higher level of tranquility than those who found their income level as insufficient. The level of tranquility was considerably higher in nurses with 11 years or more of professional experience (*p* = 0.000), 11 years or more of working experience in the organization (*p* = 0.000), and working as a manager/responsible nurse (*p* = 0.006). The type of work was found to be significant (*p* = 0.039), but there was no significant difference after the Bonferroni correction. Gender and employment institutions did not significantly differ in the tranquility scale (*p* = 0.584, *p* = 0.187).

Professional satisfaction scale analyses with independent variables are shown in Table 4. When the table is examined, income status (*p* = 0.000) and institution of employment (*p* = 0.000) made a significant difference in the total and subdimensions of the scale. In the post hoc analysis, the total score and subdimensions of professional satisfaction in nurses with insufficient income were significantly lower than the other two groups. Based on the institution of employment, the subdimension of suitability for qualifications was considerably higher in “*X*” Training and Research Hospital than in “*Y*” Training and Research Hospital. The subdimension of desire for development and the total professional satisfaction scale were significantly higher in “*X*” Training and Research Hospital than in the other two hospitals. The scale's total (*p* = 0.000) and subdimension (*p* = 0.000, *p* = 0.050) scores were significantly higher in those with 11 years or more of professional experience. Those with 11 years or more of working experience in their institution had considerably higher scores in Compliance with Qualifications (*p* = 0.003) and total professional satisfaction (*p* = 0.008), while there was no significant difference in the desire for improvement (*p* = 0.200). The total (*p* = 0.002) and subdimension scores (*p* = 0.007, *p* = 0.003) of professional satisfaction were significantly higher in those working as a manager/responsible nurse. The type of work significantly affected the desire for improvement and the total professional satisfaction scale (*p* = 0.014, *p* = 0.037). While the desire for improvement was significantly higher in daytime-only workers than nighttime-only workers, there was no significant difference between the groups after the Bonferroni correction in total professional satisfaction. Gender (*p* = 0.439, *p* = 0.430, and *p* = 0.394), educational status (*p* = 0.449, *p* = 0.058, and *p* = 0.474), and marital status (*p* = 0.100, *p* = 0.586, and *p* = 0.296) did not create a significant difference in the total and subdimensions of the professional satisfaction scale.

According to the correlation analysis, a weak positive significant relationship was found between age and professional satisfaction (*r* = 0.190, *p* < 0.001) and between age and the tranquility scale (*r* = 0.199, *p* < 0.001). There was a moderate positive correlation (*r* = 0.536, *p* < 0.001) between professional satisfaction and tranquility (Table 5).

Statistically significant differences were found between some independent variables and the dependent variable, the tranquility scale. The regression analysis was performed to evaluate the effects of these variables together. In the model, education level, income status, and professional experience explained 13.1% of the change in the total score of the tranquility scale (*F*(9,534) = 10.068; *p* < 0.001). When the variables affecting the tranquility scale were evaluated according to the beta coefficient, it was seen that income level (*β* = −0.175, −0.369), professional experience (*β* = 0.132), and educational status (*β* = 0.131) were significant predictors. It was seen that having a postgraduate education had an effect on the level of tranquility in the direction of increasing the level of tranquility compared to those with a high school education, those with more than 11 years of professional experience had an effect on the level of tranquility in the direction of increasing the level of tranquility compared to those with 10 years or less professional experience, and those who evaluated their income as insufficient or partially insufficient had an effect on the total score of the level of tranquility in the direction of decreasing the total score of the level of tranquility compared to those who evaluated their income as sufficient (Table 6).

Statistically significant differences were found between some of the independent and dependent variables of the professional satisfaction scale. The regression analysis was performed to evaluate the effects of these variables together. In the model, it was seen that tranquility score, income level, years of professional experience, and the institution of employment explained 36.6% of the change in the total score of the professional satisfaction scale (*F*(7,536) = 45.737; *p* < 0.001). A one-unit increase in the tranquility score causes a 1.16-point increase in the professional satisfaction score (*p* < 0.001). When the variables affecting the professional satisfaction scale were evaluated according to the beta coefficient, it was seen that the institution of employment (*β* = 0.232), professional experience (*β* = 0.095), and income level (*β* = −0.119) predicted significantly. It was seen that “*X*” Training and Research Hospital employees were more likely to increase professional satisfaction than “*Y*” Training and Research Hospital employees, and those with 11 years or more experience were more likely to increase professional satisfaction than those with 10 years or less experience. Those who evaluated their income as insufficient were more likely to decrease the total score of professional satisfaction than those who evaluated their income as sufficient (Table 7).

## 4. Discussion

In this study evaluating the relationship between tranquility and professional satisfaction in nurses, the average age of the participants was 31, and most of them were female. The study determined that the level of tranquility and professional satisfaction of the nurses were at a medium level. In previous studies, while the professional satisfaction levels of nurses differed between below the intermediate [28] and high [29], their level of tranquility was similarly found to be moderate [16]. The difference between the levels of professional satisfaction may be due to the physical characteristics of the institutions and the difference in professional opportunities. Because according to the literature, working environment and conditions affect professional satisfaction [23]. This study also revealed that city/institutional difference affects professional satisfaction.

The study found that nurses with postgraduate education and single nurses had higher levels of tranquility. According to the regression analysis, the level of education affected the level of tranquility in the direction of increasing it. In addition, the level of tranquility was higher in those with sufficient income. Similarly, the regression analysis also supported this. Considering that income level can be parallel to education level, the two results support each other. Being married can bring different spiritual and financial burdens. Therefore, the assumption that the material and spiritual burden is lower and the finding that income is adequate explain the higher level of tranquility among single people. Similarly, the literature found that people who perceived their income level as high had a higher level of tranquility [30]. In the study, professional experience, years of working in the organization, and working as a manager increase the level of tranquility of nurses. This situation reveals the importance of reducing turnover and organizational belonging. The study found that the mode of operation did not make a difference in the level of tranquility in nurses; however, in a case study, it was reported that nurses expressed the need for a tranquility room, especially during night shifts [31]. When the question “Do nurses working in different cities have the same tranquility?” was evaluated, it was revealed that the level of tranquility did not differ in nurses working in different cities/institutions.

Professional satisfaction, the other subject of the study, was found to be lower in those with insufficient income. The regression analysis performed in the study similarly supported this finding. A study found that nurses who reported high salaries had higher job satisfaction [32]. As stated in the literature [33], income level is one of the main factors affecting employee professional satisfaction. In this study, the factor affecting the desire for development is how nurses work. The desire for growth, a subdimension of the professional satisfaction scale, was found to be significantly higher in nurses working only during the day than in nurses working only at night. In previous studies, the results that nurses working only in daytime have higher professional/job satisfaction are similar [34, 35].

The study conducted in two cities and three hospitals found a significant city difference in professional satisfaction. Nurses working in “*X*” Training and Research Hospital reported higher levels of professional satisfaction. The institutional difference in terms of professional satisfaction was similar in the regression analysis. When the question “Do nurses working in different cities have the same level of professional satisfaction?” was evaluated, it revealed that the level of professional satisfaction differed among nurses working in different cities/institutions.

This difference may be affected by internal factors related to nurses' professional satisfaction and external factors such as working conditions, as stated in the literature [36], as well as the opportunities and living conditions provided by the city. In addition, the fact that nurses working in cities/institutions with high patient density focus more on the care process may positively affect their professional satisfaction levels. Therefore, it is recommended that the main influencing factors be examined in different studies.

The study determined that the professional satisfaction of nurses increased as professional experience and years of working in the organization increased. In one study, it was found that new graduate nurses had low levels of professional satisfaction [13]. In addition, it was revealed that nurses' professional satisfaction increased with age [28]. The studies support each other because increasing age and professional experience are parallel.

The study determined that the professional satisfaction of nurses working in managerial positions was higher. In a survey conducted with a different group, it was similarly stated that managers had higher professional satisfaction [37]. A study conducted with nurses noted that the mean satisfaction scores of nurse managers were the highest [38]. A managerial position enables the person to participate in the decision-making process and can positively affect professional satisfaction. In addition, considering that employees in managerial positions primarily work only in day shifts, it may explain the high level of professional satisfaction. Studies have shown long-term shift workers have lower professional satisfaction and higher burnout [39–41].

According to the correlation analysis, it was determined that there was a positive relationship between professional satisfaction and tranquility. The regression analysis supports this finding and shows that an increase in the level of tranquility leads to an increase in professional satisfaction. When the question “Is there a relationship between nurses' tranquility level and professional satisfaction?” of the study was evaluated, it was revealed that there was a relationship between nurses' tranquility level and professional satisfaction.

In previous studies, it has been reported that the probability of depersonalization increases as the tranquility level of health professionals decreases [42], and there is a negative relationship between depersonalization and professional satisfaction [43]. Therefore, increasing employees' tranquility should be among organizations' primary objectives.

In the correlation analysis, it was found that as age increased, both professional satisfaction and tranquility levels increased. The increase in age also means an increase in working years and professional experience. Independent variable analysis results and regression results also support this finding.

When all these results are considered, professional experience and continuity of work in the organization, which are necessary for high professional satisfaction and tranquility in nurses, come to the fore. Therefore, management practices that reduce turnover rates, such as positive workplace culture, effective communication, and decision-making participation, should be emphasized. In addition, following policies to increase the income level based on nursing will positively affect peace of mind and professional satisfaction. Finally, investigating the higher level of professional satisfaction in nurses working in metropolitan areas with different studies will contribute scientifically.

## 5. Limitations

The findings of this study were conducted in two provinces and a total of three hospitals and reflect only the participants.

## 6. Conclusion

As a result of the study, nurses' tranquility and professional satisfaction were found to be at a moderate level. The level of tranquility was significantly higher in those with postgraduate education, single people, those with adequate income, those with 11 years or more of professional experience, years of working in the organization, and those working in managerial positions.

While the desire for development was significantly higher in daytime workers, the professional satisfaction of nurses with adequate income level, professional experience, and working years in the institution 11 years or more, managerial position, and working in “*X*” Training and Research Hospital was found to be significantly higher.

A positive relationship was found between tranquility and professional satisfaction. Similarly, the regression result showed that an increase in the level of tranquility affected professional satisfaction in the direction of increasing it. In this direction, increasing tranquility will be an essential approach to increase professional satisfaction in nurses.

## 7. Implications for Future Nursing Management Research

Nurses, who take an active role in the provision of quality health services, are the professional group of the team with indisputable importance. In this context, tranquility and professional satisfaction, which were evaluated at a moderate level in the study, should be handled more carefully by institutions. This study, which evaluates these two essential concepts together, fills the gap in the scientific field and reveals the positive effect of professional experience and long-term work in the institution on tranquility and professional satisfaction. Regulation of working environments affects nurses' levels of tranquility, and regular implementation of practices such as recognition and promotion will increase professional satisfaction and tranquility and will positively reflect on the quality of care.

## Figures and Tables

**Table 1:** Distribution of demographic data of participants.

		*n*	%
Age	*X* ± sd = 31.49 ± 7.54		

Gender	K	454	83.2
	E	92	16.8

Education status	^∗^High school	21	3.8
	Associate and undergraduate	496	90.8
	Postgraduate	29	5.3

Marital status	Married	259	47.4
	Single	287	52.6

Income status	Adequate	127	23.3
	Partially sufficient	290	53.1
	Inadequate	129	23.6

Working organization	“*Y*” Training and Research Hospital	205	37.5
	“*Z*” State Hospital	165	30.2
	“*X*” Training and Research Hospital	176	32.3

Years of professional expression	0–10 years	375	
	Over 11 years	171	31.3

Duration of employment in the organization	0–10 years	414	75.8
	Over 11 years	132	24.2

Working position	Manager/responsible nurse	74	13.6
	Nurse	472	86.4

Mode of operation	Day alone	114	20.9
	Lonely night	2	0.4
	Night and day	430	78.8

^∗^High school; nontechnician high school graduate nurse.

**Table 2:** Participants' tranquility scale, professional satisfaction scale, and subscale scores.

	*n*	Min	Max.	Mean	Std. deviation
Tranquility scale	546	8	52	28.10	5.20
Professional satisfaction scale total	546	25	93	59.71	11.59
Compliance with Qualifications	546	15	65	38.93	8.70
Willingness to Evolve	546	9	31	20.78	3.99

**Table 3:** Tranquility scale analysis according to independent variables.

	*n*	*X* ± sd	*t*/*F*^∗^	*p*
Gender				
K	454	28.04 ± 5.10	−0.584	0.584
E	92	28.37 ± 5.66		
Education status				
High school	21	25.81 ± 5.49	4.093^∗^	0.017
Associate and undergraduate	496	28.08 ± 5.19		
Postgraduate	29	30.03 ± 4.46		
Marital status				
Married	259	27.47 ± 5.27	−2.679^∗^	0.008
Single	287	28.66 ± 5.07		
Income status				
Adequate	127	30.06 ± 4.59	23.858^∗^	0.000
Partially sufficient	290	28.27 ± 4.51		
Inadequate	129	25.78 ± 6.25		
Working organization				
“*Y*” Training and Research Hospital	205	27.72 ± 5.39	1.683	0.187
“*Z*” State Hospital	165	28.70 ± 4.59		
“*X*” Training and Research Hospital	176	27.98 ± 5.47		
Years of professional expression				
0–10 years	375	27.46 ± 5.08	−4.330^∗^	0.000
Over 11 years	171	29.50 ± 5.26		
Duration of employment in the organization				
0–10 years	414	27.65 ± 5.03	−3.634^∗^	0.000
Over 11 years	132	29.52 ± 5.48		
Working position				
Manager/responsible nurse	74	29.65 ± 5.34	2.774^∗^	0.006
Nurse	472	27.86 ± 5.14		
Mode of operation				
Day alone	114	29.11 ± 5.04	3.272^∗^	0.039
Lonely night	2	24.00 ± 11.31		
Night and day	430	27.85 ± 5.19		

**Table 4:** Analyses of professional satisfaction scale and subscales according to independent variables.

	Compliance with qualifications			Willingness to evolve			Total professional satisfaction		
	*X* ± sd	*t*/*F*^∗^	*p*	*X* ± sd	*t*/*F*^∗^	*p*	*X* ± sd	*t*/*F*^∗^	*p*
Gender									
K	39.06 ± 8.67	0.774	0.439	20.84 ± 3.93	0.790	0.430	59.90 ± 11.45	0.853	0.394
E	38.29 ± 8.86			20.48 ± 4.29			58.77 ± 12.27		
Education status									
High school	36.71 ± 7.24	0.803^∗^	0.449	20.19 ± 3.07	2.864^∗^	0.058	56.90 ± 9.57	0.747^∗^	0.474
Associate and undergraduate	39.06 ± 8.69			20.71 ± 4.00			59.77 ± 11.55		
Postgraduate	38.34 ± 9.75			22.45 ± 4.14			60.79 ± 13.45		
Marital status									
Married	38.29 ± 8.44	−1.646	0.100	20.88 ± 4.05	0.545	0.586	59.17 ± 11.40	−1.047	0.296
Single	39.52 ± 8.90			20.69 ± 3.94			60.21 ± 11.75		
Income status									
Adequate	41.26 ± 8.81	13.543^∗^	0.000	21.45 ± 3.82	10.387^∗^	0.000	62.71 ± 1132	14.953^∗^	0.000
Partially sufficient	39.29 ± 8.43			21.09 ± 3.87			60.38 ± 11.20		
Inadequate	35.84 ± 8.70			19.43 ± 4.13			55.26 ± 11.50		
Working organization									
“Y” Training and Research Hospital	37.38 ± 9.07	7.915^∗^	0.000	19.99 ± 3.92	12.229^∗^	0.000	57.37 ± 12.01	11.018^∗^	0.000
“Z” State Hospital	38.78 ± 8.14			20.52 ± 3.74			59.30 ± 10.39		
“X” Training and Research Hospital	40.89 ± 8.43			21.94 ± 4.06			62.82 ± 11.50		
Years of professional expression									
0–10 years	37.84 ± 8.35	−4.423	0.000	20.55 ± 3.97	−1.966	0.050	58.39 ± 11.18	−3.996	0.000
Over 11 years	41.33 ± 8.99			21.27 ± 4.00			62.61 ± 11.96		
Duration of employment in the organization									
0–10 years	38.31 ± 8.61	−2.969	0.003	20.65 ± 3.96	−1.283	0.200	58.97 ± 11.45	−2.671	0.008
Over 11 years	40.88 ± 8.71			21.17 ± 4.07			62.05 ± 11.74		
Working position									
Manager/repo sensible nurse	41.46 ± 9.06	0.683	0.007	22.04 ± 3.72	2.944	0.003	63.50 ± 11.50	3.046	0.002
Nurse	38.54 ± 8.58			20.58 ± 4.00			59.12 ± 11.50		
Mode of operation									
Day alone	40.19 ± 8.15	2.284^∗^	0.103	21.72 ± 4.13	4.309^∗^	0.014	61.91 ± 10.91	3.316^∗^	0.037
Lonely night	31.00 ± 1.41			18.50 ± 2.12			49.50 ± 0.707		
Night and day	38.93 ± 8.82			20.54 ± 3.93			59.18 ± 11.71		

**Table 5:** Correlation analysis between age, tranquility scale, and professional satisfaction scale.

		Total professional satisfaction	Tranquility scale
Age	*r*	0.190^∗∗^	0.199^∗∗^
Total professional satisfaction	*r*	1	0.536^∗∗^

^∗^ *p* < 0.05.

^∗∗^ *p* < 0.001.

**Table 6:** The effect of independent variables on the tranquility scale: the regression analysis.

	*B*	*β*	*t*	*p*	95% CI for *B*		Zero-order	Partial	Part
Constant	27.180		24.113	0.000	24.966	29.394			
License	1.724	0.099	1.637	0.102	−0.345	3.792	−0.011	0.071	0.065
Postgraduate	2.932	0.131	2.160	0.031	0.265	5.599	0.092	0.093	0.086
Married	0.501	0.050	1.078	0.282	−0.412	1.413	0.134	0.047	0.043
Income is partially sufficient	−1.768	−0.175	−3.521	0.000	−2.755	−0.782	0.052	−0.151	−0.141
Insufficient income	−4.375	−0.369	−7.370	0.000	−5.541	−3.209	−0.277	−0.304	−0.295
11 years or more of professional experience	1.432	0.132	2.791	0.005	0.424	2.439	0.190	0.120	0.112
Manager/responsible nurse	0.782	0.053	1.200	0.231	−0.498	2.063	0.123	0.052	0.048
“Z” state hospital	0.586	0.054	1.180	0.238	−0.389	1.561	0.079	0.051	0.047
“X” Training and Research Hospital	0.504	0.047	1.029	0.304	−0.458	1.466	−0.034	0.044	0.041

**Table 7:** The effect of independent variables on the professional satisfaction scale: the regression analysis.

	*B*	*β*	*t*	*p*	95% CI for *B*		Zero-order	Partial	Part
Constant	25.750		9.917	0.000	20.649	30.851			
Tranquility score	1.158	0.514	13.994	0.000	0.996	1.321	0.560	0.517	0.478
Income is partially sufficient	−0.904	−0.039	−0.912	0.362	−2.851	1.044	0.054	−0.039	−0.031
Insufficient income	−3.223	−0.119	−2.650	0.008	−5.612	−0.834	−0.216	−0.114	−0.091
11 years or more of professional experience	2.345	0.095	2.492	0.013	0.496	4.193	0.178	0.107	0.085
Manager/responsible nurse	0.701	0.021	0.560	0.576	−1.759	3.160	0.128	0.024	0.019
“Z” State Hospital	0.410	0.016	0.424	0.672	−1.492	2.312	−0.029	0.018	0.014
“X” Training and Research Hospital	5.710	0.232	5.991	0.000	3.838	7.582	0.187	0.251	0.205

## Data Availability

The data used to support the findings of this study are available from the corresponding author upon reasonable request.

## References

[1] van der Cingel M., Brouwer J. (2021). What Makes a Nurse Today? A Debate on the Nursing Professional Identity and Its Need for Change. *Nursing Philosophy*.

[2] Biganeh J., Ashtarinezhad A., Behzadipour D., Khanjani N., Tavakoli Nik A., Bagheri Hosseinabadi M. (2022). Investigating the Relationship Between Job Stress, Workload and Oxidative Stress in Nurses. *International Journal of Occupational Safety and Ergonomics*.

[3] Demirci İ., Ekşi H. (2017). Development of Peace Scale and Examination of Its Psychometric Properties. *Journal of Values Education*.

[4] Christoffersen V. R., Škodlar B., Henriksen M. G. (2022). Exploring Tranquility: Eastern and Western Perspectives. *Frontiers in Psychology*.

[5] (2023). Tureng. https://tureng.com/tr/turkce-ingilizce/tranquility.

[6] Reding N. (2022). The Living Experience of Feeling Peaceful. *Nursing Science Quarterly*.

[7] Aruasa W. K., Chirchir L. K., Chebon S. K. (2019). Determinants of Physicians and Nurses’ Professional Satisfaction at the Moi Teaching and Referral Hospital. *Journal of Health, Medicine and Nursing*.

[8] Farman A., Kousar R., Hussain M., Waqas A., Gillani S. A. (2017). Impact of Job Satisfaction on Quality of Care Among Nurses on the Public Hospital of Lahore, Pakistan. *Saudi Journal of Medical and Pharmaceutical Sciences*.

[9] Wijn A. N., Fokkema M., Doef M. P. (2022). The Prevalence of Stress-Related Outcomes and Occupational Well-Being Among Emergency Nurses in the Netherlands and the Role of Job Factors: A Regression Tree Analysis. *Journal of Nursing Management*.

[10] Saeidi R., İzanloo A., İzanlou S. (2020). A Study of the Relationship Between Job Satisfaction and Burnout Among Neonatal Intensive Care Unit Staff. *Iranian Journal of Neonatology*.

[11] Geese F., Zwakhalen S., Lucien B., Hahn S. (2022). Job Satisfaction of Advanced Practice Nurses in Cancer Care: A Systematic Review. *European Journal of Oncology Nursing*.

[12] Taşkaya S., Aksoy A. (2021). A Bibliometric Analysis of Workplace Incivility in Nursing. *Journal of Nursing Management*.

[13] Ulupinar S., Aydogan Y. (2021). New Graduate Nurses’ Satisfaction, Adaptation and Intention to Leave in Their First Year: A Descriptive Study. *Journal of Nursing Management*.

[14] Demirci İ., Ekşi H. (2018). Keep Calm and Be Happy: A Mixed Method Study From Character Strengths to Well-Being. *Educational Sciences: Theory and Practice*.

[15] Sundar T. K. B., Sargenius H., Kvarme L. G., Sparboe-Nilsen B. (2024). Norwegian Pre-Service Teacher Students’ and Public Health Nursing Students’ Views on Health: A Qualitative Study of Students’ Perceptions. *International Journal of Qualitative Studies on Health and Well-Being*.

[16] Gumusler Basaran A., Kefeli Col B., Genc Kose B. (2024). Evaluation of the Relationship Between the Levels of Patience and Tranquillity and Conflict Resolution Styles of Executive Nurses. *Journal of Nursing Management*.

[17] Moore A., Thal W. (2021). Traits of the Resilient Nurse. *Nursing Made Incredibly Easy*.

[18] Chakraborty D., Biswas W. (2021). Think Love, Think Peace, Think Harmony: Rethinking on Industrial Tranquility. *Business Perspectives and Research*.

[19] Tūtlys V., Gedvilienė G., Didžiulienė R. (2021). The Influence of Teacher Professional Burnout on Professional Satisfaction in Professional Career Development. *The New Educational Review*.

[20] Specchia M. L., Cozzolino M. R., Carini E. (2021). Leadership Styles and Nurses’ Job Satisfaction. Results of a Systematic Review. *International Journal of Environmental Research and Public Health*.

[21] Ozdoba P., Dziurka M., Pilewska-Kozak A., Dobrowolska B. (2022). Hospital Ethical Climate and Job Satisfaction Among Nurses: A Scoping Review. *International Journal of Environmental Research and Public Health*.

[22] Penconek T., Tate K., Bernardes A. (2021). Determinants of Nurse Manager Job Satisfaction: A Systematic Review. *International Journal of Nursing Studies*.

[23] Gil M. F. H., Hernández J. A. R., Ibáñez-López F. J. (2022). Relationship Between Job Satisfaction and Workload of Nurses in Adult Inpatient Units. *International Journal of Environmental Research and Public Health*.

[24] Işikli A. G., Şen H., Soydaş D. (2021). Assessment of Psychological Resilience, Job Satisfaction, and Fear Level of Nurses Infected and Not Infected With the COVID-19 Virus. *Journal of Psychiatric Nursing*.

[25] Mardanov I. (2021). Intrinsic and Extrinsic Motivation, Organizational Context, Employee Contentment, Job Satisfaction, Performance and Intention to Stay. *Evidence-Based HRM: A Global Forum for Empirical Scholarship*.

[26] Erdal N. (2022). Investigation of the Effect of Job Satisfaction and Organizational Citizenship Behavior on Trust in the Manager in the Example of Nurses and Health Technicians. *Asian Journal of Advances in Research*.

[27] Kuzgun Y., Aydemir Sevim S., Harmanci Z. (1999). Developing a Job Satisfaction Scale. *Turkish Psychological Counseling and Guidance Journal*.

[28] Kaya Ş. D., Mehmet N., Şafak K. (2022). Professional Commitment, Satisfaction and Quality of Life of Nurses During the Covid-19 Pandemic in Konya, Turkey. *Ethiopian Journal of Health Sciences*.

[29] Savitsky B., Shvartsur R., Findling Y., Ereli A., Hendel T. (2023). Unveiling the Components of Professional Satisfaction Among Novice Nurses. *Israel Journal of Health Policy Research*.

[30] Çelik O. B. (2020). Investigation of Tranquility Levels of University Students in Terms of Various Variables. *International Journal of Contemporary Educational Studies*.

[31] Kennedy Oehlert J. A., Bowen C. M., Wei H., Leutgens W. (2022). Tranquility Rooms for Team Member Well-Being: Implementation During COVID-19 Pandemic. *Patient Experience Journal*.

[32] Kootahi Z. E., Yazdani N., Parsa H., Erami A., Bahrami R. (2023). Professional Values and Job Satisfaction Neonatal Intensive Care Unit Nurses and Influencing Factors: A Descriptive Correlational Study. *International Journal of Africa Nursing Sciences*.

[33] Bhardwaj A., Mishra S., Kumar Jain T. (2021). An Analysis to Understanding the Job Satisfaction of Employees in Banking Industry. *Materials Today: Proceedings*.

[34] Koyuncu O., Arslan S. (2022). Levels of Vocational Satisfaction, Burnout and Compassion Fatigue of Health Professionals Working in Pediatric Clinics. *World Journal of Clinical Pediatrics*.

[35] Dodia P., Parashar N. (2020). Shift-Work Job Stress, Psychological Distress, and Job Satisfaction Among Employees. *The International Journal of Indian Psychology*.

[36] Yasin M., Kerr M. S., Wong C. A., Bélanger C. H. (2020). Factors Affecting Nurses’ Job Satisfaction in Rural and Urbanacute Care Settings: A PRISMA Systematic Review. *Journal of Advanced Nursing*.

[37] Demirhan M., Ak E., Şanser E., Buğu E., Bağ N., Öncü M. E. (2024). Examining the Relationship Between Professional Satisfaction and Job Performance of Managers. *National Journal of Social and Educational Sciences*.

[38] Barmanpek N. K., Sahin A., Demirel C., Parlar Kiliç S. (2022). The Relationship Between Nurses’ Job Satisfaction Levels and Quality of Life. *Perspectives in Psychiatric Care*.

[39] Dall’Ora C., Griffiths P., Ball J., Simon M., Aiken L. H. (2015). Association of 12 h Shifts and Nurses’ Job Satisfaction, Burnout and Intention to Leave: Findings From a Cross-Sectional Study of 12 European Countries. *BMJ Open*.

[40] Ruiz-Fernández M. D., Pérez-García E., Ortega-Galán Á.M. (2020). Quality of Life in Nursing Professionals: Burnout, Fatigue, and Compassion Satisfaction. *International Journal of Environmental Research and Public Health*.

[41] Banakhar M. (2017). The Impact of 12-Hour Shifts on Nurses’ Health, Wellbeing, and Job Satisfaction: A Systematic Review. *Journal of Nursing Education and Practice*.

[42] Ferraz J. A. d. C., Zanin L., Oliveira A. M. G., Flório F. M. (2023). Prevalência e Fatores Associados à Síndrome de Burnout em Profissionais da Saúde Indígena no Brasil. *Ciência & Saúde Coletiva*.

[43] Shalaby R., Oluwasina F., Eboreime E. (2023). Burnout Among Residents: Prevalence and Predictors of Depersonalization, Emotional Exhaustion and Professional Unfulfillment Among Resident Doctors in Canada. *International Journal of Environmental Research and Public Health*.

